# Quantification of whole-body and segmental skeletal muscle mass using phase-sensitive 8-electrode medical bioelectrical impedance devices

**DOI:** 10.1038/ejcn.2017.27

**Published:** 2017-03-22

**Authors:** A Bosy-Westphal, B Jensen, W Braun, M Pourhassan, D Gallagher, M J Müller

**Affiliations:** 1 Institut für Ernährungsmedizin, Universität Hohenheim, Stuttgart, Germany; 2 Institut für Humanernährung und Lebensmittelkunde, Christian-Albrechts-University Kiel, Kiel, Germany; 3 Seca Gmbh & Co. KG., Hamburg, Germany; 4 Body Composition Unit, New York Obesity Nutrition Research Center, St Luke’s-Roosevelt Hospital, New York, NY, USA

## Abstract

**Background/Objectives:**

Bioelectrical impedance analysis (BIA) provides noninvasive measures of skeletal muscle mass (SMM) and visceral adipose tissue (VAT). This study (i) analyzes the impact of conventional wrist-ankle vs segmental technology and standing vs supine position on BIA equations and (ii) compares BIA validation against magnetic resonance imaging (MRI) and dual X-ray absorptiometry (DXA).

**Subjects/Methods:**

One hundred and thirty-six healthy Caucasian adults (70 men, 66 women; age 40±12 years) were measured by a phase-sensitive multifrequency BIA (seca medical body composition analyzers 515 and 525). Multiple stepwise regression analysis was used to generate prediction equations. Accuracy was tested vs MRI or DXA in an independent multiethnic population.

**Results:**

Variance explained by segmental BIA equations ranged between 97% for total SMM_MRI_, 91–94% for limb SMM_MRI_ and 80–81% for VAT with no differences between supine and standing position. When compared with segmental measurements using conventional wrist-ankle technology. the relationship between measured and predicted SMM was slightly deteriorated (*r*=0.98 vs *r*=0.99, *P*<0.05). Although BIA results correctly identified ethnic differences in muscularity and visceral adiposity, the comparison of bias revealed some ethnical effects on the accuracy of BIA equations. The differences between LST_DXA_ and SMM_MRI_ at the arms and legs were sizeable and increased with increasing body mass index.

**Conclusions:**

A high accuracy of phase-sensitive BIA was observed with no difference in goodness of fit between different positions but an improved prediction with segmental compared with conventional wrist-ankle measurement. A correction factor for certain ethnicities may be required. When compared with DXA MRI-based BIA equations are more accurate for predicting muscle mass.

## Introduction

Skeletal muscle mass (SMM) is an important clinical target parameter, for example, in patients with chronic obstructive pulmonary disease, chronic glucocorticoid therapy, sarcopenia, frailty, cancer cachexia and others.^1^ Noninvasive and inexpensive measurement of total and regional skeletal muscle is therefore of great practical importance. In this context, whole-body magnetic resonance imaging (MRI) and dual X-ray absorptiometry (DXA) are considered as valid reference methods. However, the use of more simple and inexpensive bioelectrical impedance analysis (BIA) has become more and more popular.

The validity of BIA has been investigated in numerous studies using appendicular lean soft tissue (LST=bone-free fat-free mass) by DXA as a reference.^2, 3, 4, 5, 6, 7, 8, 9, 10, 11^ The accuracy of SMM_BIA_ has been confirmed in all but one of these studies.^9^ The use of LST as a proxy for SMM is however not without limitation. This is evident from Swiss reference data showing an increase in fat mass index (kg/m^2^) with advancing age, whereas fat-free mass index (kg/m^2^) remained fairly constant across age groups.^12^ Because the decrease in muscle mass with age is likely compensated by an increase in connective tissue (i.e. an increase in the fat-free mass component of adipose tissue,^13^ fat-free mass index is insensitive to age-related changes in muscle mass. In addition, comparison of regional LST measured by DXA with SMM assessed by the gold standard MRI revealed that the contribution of skeletal muscle to appendicular LST is also lower at a higher degree of adiposity, especially in women who store more adipose tissue at the limbs compared with men who proportionally store more adipose tissue at the trunk with increasing FMI.^14^ Thus, with advancing age and adiposity the increase in connective tissue can mask a decrease in SMM at unchanged total LST or fat-free mass. In addition, the BIA assumptions of a constant hydration are violated with increasing adiposity because of the higher ratio of extracellular to intracellular water in the adipose tissue part of the connective tissue.^15^ Validation studies using state of the art whole-body imaging technology as a reference to validate SMM_BIA_ are scarce.^16, 17^ In the study by Janssen *et al.*^17^ equations for predicting SMM of Caucasian subjects were derived from conventional wrist-ankle BIA measurements at 50 kHz on the right side of the body. The Caucasian-derived equation was subsequently applied to a multiethnic sample and found to be accurate for Hispanics and African-Americans, but it underestimated SMM in Asians.^17^ A BIA prediction equation could therefore be influenced by ethnicity that determines the shape, relative muscularity and length of the trunk, arms and legs. Compared with conventional whole-body wrist-ankle methods, BIA measurements with a so-called ‘8-electrode system’ use a 4-electrode setup in different arrangements around the body and thus allow a segmental analysis of body composition. These systems may therefore compensate for ethnic differences in body shape (e.g. length and muscularity of extremities compared with the trunk) and may therefore be more accurate than BIA measurements based on conventional wrist-ankle technology with four electrodes only. In addition, non-phase-sensitive devices that only measure the absolute value of the impedance (|*Z*|) could perform worse because they do not provide an output for reactance (Xc) that can be a valuable predictor of muscle mass because Xc is related to the number and composition of cells. The seca medical body composition analyzers (mBCA) 515 and 525 are phase-sensitive 8-electrode medical BIA devices that cover a full range of frequencies from 1 to 500 kHz and allow segmental analysis of the whole body.^18^

The aims of the present study were (i) to compare goodness of fit for BIA equations that predict SMM and visceral fat and are derived (a) from conventional wrist-ankle vs segmental impedance measurements and (b) from supine vs standing positions (seca mBCA 515 vs 525). In addition, the aim was (ii) to validate BIA results (a) vs total and regional SMM by MRI and (b) vs LST by DXA in an independent multiethnic population with a wide variety of limb and trunk muscularity.

## Subjects and methods

Details of this study protocol were described previously.^18^ In the first part of the study BIA equations for prediction of SMM_MRI_ and VAT_MRI_ were generated and compared between (a) conventional wrist-ankle vs segmental technology (seca mBCA 525) or (b) standing vs supine position (seca mBCA 515 vs 525). In addition, BIA equations for prediction of LST_DXA_ were generated using segmental measurements in the supine position (seca mBCA 525). Owing to commercial sensitivities, the details of the equations are not available for publication. A total of 136 Caucasian men and women (body mass index (BMI) 20.0–34.7 kg/m^2^) aged 18–65 years were recruited from the area of Kiel, Germany.

The second part of the study was to validate the developed equations for SMM, VAT and LST in an independent multiethnic sample of 123 men and women (BMI 18.7–34.4 kg/m^2^) aged 18–65 years (32 Caucasians, 31 Asians, 30 Afro-Americans and 30 Hispanics) who were recruited at the New York Obesity Nutrition Research Center, USA.

Exclusion criteria for study participation were: acute and chronic diseases (especially hypertension, renal and cardiac insufficiency), regular intake of medications (except for contraceptives), amputation of limbs, electrical implants as cardiac pacemaker, metallic implants (except tooth implants), pregnancy or breastfeeding period, current alcohol abuse and extensive tattoos at arms or legs. Edema of ankles was excluded by inspection (and manual compression if appropriate). The study was approved by the ethics committees of the Christian-Albrechts-University Kiel and St Luke’s-Roosevelt Hospital, New York. All subjects provided their fully informed and written consent before participation.

The subjects were asked to come to the study centers between 0700 and 0730 hours. BIA and DXA measurements were taken in the morning after an overnight fast. Whole-body MRI measurements took place at a separate appointment not more than 4 days apart.

### Anthropometrics

Body height and weight were obtained on the measuring station seca 285 with an accuracy of ±50 g up to 100 kg for the scale and ±2 mm for the stadiometer. Waist circumference was measured midway between the lowest rib and the uppermost boarder of the iliac crest in the medial axillary line and at the end of normal expiration using a non-stretchable tape (circumference measuring tape seca 201).

### Bioelectrical impedance analysis

The seca mBCA 515 and 525 use four pairs of electrodes (eight electrodes in total) that are positioned at each hand and foot. The 8-electrode technique enables segmental impedance measurement of the arms and legs. Impedance is measured with a current of 100 μA at frequencies between 1 and 1 000 kHz. The mBCA 515 is designed for measurements in the standing position and consists of a platform with an integrated scale, a handrail system and a display and operation unit. Each side of the ascending handrail carries six electrodes of which two were chosen depending on person’s height. According to the manufacturer´s instructions, arms should be held straight.

The mBCA 525 is designed for measurements in the supine position and can be operated using either four adhesive electrodes on the right side of the body or eight electrodes on both sides while the subject is lying on a non-conductive surface. Adhesive gel electrodes (Kendall, H59P, Covidien IIc, Mansfield, MA, USA) were placed at defined anatomical sites on the dorsal surfaces of the hand, wrist, ankle and foot according to the manufacturer’s instructions as follows: the proximal edge of the first electrode was attached from an imaginary line at styloid process of the ulna and the distal edge of the finger electrode on an imaginary line from the middle of the metacarpophalangeal joints of the index and middle fingers. The distal edge of the toe electrode was placed from an imaginary line through the middle of the metatarsophalangeal joints of the second and third toes. The proximal edge of the ankle joint electrode was attached along a line through the highest points of the outer and inner ankle bones.

Participants were asked not to exercise within 12 h and drink alcohol within 24 h before the impedance measurement.

The duration of each BIA measurement was 75 s. Measurements in the supine position were taken after lying down for 10 min. Resistance (*R*) and reactance (Xc) values obtained at 50 kHz frequencies were used for generation of the prediction equations. Impedance index was calculated as height^2^/*R*. Two indices, Index*R*_50 trunk/extremities_ and IndexXc_50 trunk/extremities_ were derived from segmental *R* and Xc values (means of left and right body side) to represent the relative contribution of trunk and extremities to total body conductivity and thus to correct for differences in body shape.^18^ All measurements in each study center (Kiel and New York) were performed by the same investigator.

Intra- and interoperator reproducibility for total SMM_BIA_ calculated as coefficient of variation from three replicate measurements by two observers in four participants (BMI 23.8±1.3 kg/m^2^) were 0.4 and 0.6%.

### Dual X-ray absorptiometry

A whole-body DXA scan was performed to measure appendicular LST using a Hologic Discovery A densitometer and the whole-body software 12.6.1:3 (Hologic Inc., Bedford, MA, USA) in the Kiel study centre and an iDXA Software (version 11.4; GE Lunar, Madison, WI, USA) in New York.

### Magnetic resonance imaging

*In Kiel*: Measurements of SMM and visceral adipose tissue (VAT) volumes were performed in a supine position with arms extended above the heads using a Magnetom Avanto 1.5-T scanner (Siemens Medical Systems, Erlangen, Germany). The entire body from wrist to ankle was scanned using continuous axial images with 8 mm slice thickness and 2 mm interslice gaps. Images were obtained using a T1-weighted gradient-echo sequence (TR 157 ms, TE 4 ms, flip angle 70°, voxel size 3.9 × 2 × 8 mm^3^). For measurements at the trunk participants were required to hold their breath. All images were segmented manually using Slice-O-Matic, Tomovision 4.3 Software (Montreal, QC, Canada). VAT was evaluated in slices from the diaphragm (top of the liver or the base of the lungs, T10) to the femur heads. The software employed knowledge-based image processing to label pixels as fat and nonfat components using a threshold for adipose tissue on the basis of the gray-level histograms of the images. Each slice was manually reviewed and voxels arising from fatty bowel content were deleted. Total SMM (excluding head and neck) and VAT were determined from the sum of all tissue areas (cm^2^) multiplied by the slice thickness. Coefficients of variation for repeated measurements of SMM and VAT were 1.8 and 1.5%.

*In New York*: Total body skeletal muscle (SMM) and VAT were measured using whole-body multislice MRI as described previously.^19, 20^ Subjects were placed on a 1.5 T scanner platform (GE 6X Horizon; GE Healthcare Milwaukee, WI, USA) with their arms extended above their heads. The protocol involved the acquisition of ≈40 axial images, 10 mm in thickness and at 40 mm intervals across the whole body. SliceOmatic Image Analysis Software (version 4.2; Tomovision, Montreal, QC, Canada) was used to analyze images on a PC workstation (Gateway, Madison, WI, USA). MRI-volume estimates were converted to mass using the assumed density of 1.04 kg/l for skeletal muscle and 0.92 kg/l for adipose tissue.^21^ All scans in this study were read by the same technician at the New York Obesity Nutrition Research Center. The technical error (coefficients of variation) for three repeated readings of the same scan by the same analyst for skeletal muscle and VAT were 2.4% and 1.97%, respectively.

### Statistics

Data analyses were performed with R Software (version 3.0.1; R Foundation, Vienna, Austria). Descriptive statistics are presented as means±s.d. Differences between independent samples (e.g. men and women or ethnic groups) were analysed using unpaired *t*-test. Differences between parameters of body composition assessed by BIA and DXA or MRI were tested using paired *t*-test. Pearson’s correlation coefficient was calculated for relationships between variables and compared according to Eid *et al.*^22^ A *P*-value <0.05 was considered significant.

### Development and comparison of BIA algorithms

Stepwise multiple-regression analysis was applied to the data of the Caucasian subjects to derive the best-fitting regression equations to predict total and segmental SMM_MRI_ and LST_DXA_ from conventional wrist-ankle and segmental impedance data obtained in a standing or supine position. Goodness of fit was assessed by determination coefficient (*R*^2^, proportion of the total variance in the dependent variable that is explained by the independent variables) and precision by the pure error. The pure error was calculated as the root mean square of the differences between predicted and measured data (the smaller the pure error, the greater the precision of the tested equation). The impedance index at 50 kHz was used as the primary independent variable, and then secondary variables were tested as additional predictors (*R* and Xc at 50 kHz, weight, age, sex and the two indices for segmental impedance and waist circumference in the case of VAT prediction). Optimal combination of predictor variables was selected by consideration of the correlation between predicted and observed data (which should be maximized). The prediction accuracy was compared between BIA equations derived from the supine vs standing and conventional wrist-ankle vs segmental measurements and was tested by comparison of correlation coefficients.^22^

### Validation of BIA equations vs MRI and DXA in an independent multiethnic sample

To determine the effect of ethnicity on the accuracy of the BIA prediction equation, the equations derived from the Caucasian subjects were applied to the Caucasian, Afro-American, Asian and Hispanic subjects in an independent population from New York. Analysis according to Bland and Altman was used to determine absolute agreement between the body composition assessed by criterion methods (MRI and DXA) and BIA. According to this approach, the bias is the difference between measured and predicted values of SMM or LST and the error is the standard deviation of the bias.^23^ The dependency of the bias on the mean of measured and predicted values was tested using correlation analysis. The limits of agreement, calculated as bias±2 s.d. error (i.e. 95% confidence interval of the individual difference), were used to test agreement between the two methods (measured and predicted SMM values). The validity of BIA results vs MRI and DXA was compared between different ethnic groups by comparison of the bias.

The pure error (accuracy) statistic was used for cross-validation of BIA results (i.e. testing the predictive power of the BIA equation for data not used in the equation’s development). The pure error was calculated as the root mean square of the differences between predicted and measured data (the smaller the pure error, the greater the accuracy of the tested equation).


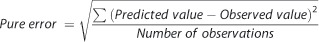


## Results

Basic characteristics of the Caucasian study population used to generate the BIA equations are shown in Table 1 stratified by sex. The prevalence of normal weight, overweight and obesity was 57%, 32% and 11%, respectively. Men had a higher BMI and waist circumference compared with women. Accordingly, impedance raw data obtained in the supine position with segmental measurements were lower in men. Besides these impedance data, weight, age and gender were used as independent variable in the equations for total SMM. For prediction of SMM of the arms and legs information on segmental impedance of the limbs was used. In addition to impedance data of the trunk, equations for prediction of VAT were obtained using waist circumference, waist circumference^2^, age, height, weight and the interaction terms waist circumference × sex and waist circumference^2^ × sex as predictors. Results of the stepwise regression analyses with total and limb SMM_MRI_ and VAT_MRI_ as the dependent variables are provided in Table 5.

In Table 2 goodness of fit for BIA equations obtained from measurements in the standing and supine position using segmental or conventional wrist-ankle measurements are described by coefficient of determination and precision is given by the pure error. Ninety-seven percent of the variance in total SMM_MRI_ and 80–81% of the variance in VAT were explained by BIA equations obtained from measurements in the supine and standing position. There was also no difference between the variance in SMM_MRI_ of the arms and legs explained by BIA in the two positions. By contrast, the variance in SMM_MRI_ explained by BIA measurements using conventional wrist-ankle measurements on the right side of the body was significantly less when compared with the segmental measurement on both sides of the body in the supine position as a reference (*r*=0.98 vs *r*=0.99, *P*<0.01).

When compared with the prediction of SMM, the accuracy of VAT prediction by BIA was considerably lower and waist circumference and gender were major predictors with impedance data having only a minor contribution to the prediction of the algorithm (Table 5).

Basic characteristics of the multiethnic population used to validate the BIA equations are given in Table 3 stratified by ethnic group and gender. Age ranged from 18 to 65 years and BMI from 18.7 to 34.4 kg/m^2^ with similar values between ethnic groups. Table 4 shows mean values for SMM, LST and VAT measured by the reference methods and predicted from BIA and compares (i) results from BIA vs the reference method within each ethnic group and (ii) the validity of SSM and LST predicted from BIA equations between different ethnic groups. When compared with Caucasians, Asians had lower SMM of the total body, arms and legs. Hispanics had a lower and Afro-Americans a higher SMM of the legs. VAT results from MRI were higher in Hispanics and lower in Afro-Americans when compared with Caucasians. Although the mean bias for prediction of total and regional SMM and LST was low in all ethnic groups, it was significant for all LST results and most SMM compartments, with exception of arm SMM in Asians and Hispanics and total and leg SMM in Afro-Americans. The bias for VAT prediction was significant in Asians and Hispanics only. Comparison of the bias between Caucasians and different ethnic groups revealed a significant ethnic effect on the accuracy of BIA prediction equations (i) for VAT in Asians and Hispanics and (ii) for total and leg SMM and LST of the arms and legs for Afro-Americans and (iii) for leg SMM in Hispanics.

The differences between limb LST_DXA_ and SMM_MRI_ predicted by BIA ranged between 1.5 and 7.3 kg for arms and legs, respectively. The difference between LST_DXA_ and SMM_MRI_ was higher in men compared with women (data not shown, *P*<0.001) and increased with increasing BMI (*r*=0.37, *P*<0.001) in the total multiethnic population.

## Discussion

The present study investigated the impact of a standing vs supine position and conventional wrist-ankle vs segmental measurements on the goodness of fit of a BIA equation for prediction of SMM and VAT. As a result, a high proportion of the variance in total (97%) and limb SMM_MRI_ was explained by BIA equations with no difference in the goodness of fit between BIA measurements in the standing and supine position. This result is important because it refutes the common belief that BIA analyzers that incorporate foot and hand contact points for standing on four metal plates and holding a rod with the fingers and thumb of each hand (e.g. from seca, Tanita, Omron or BioSpace) have a significant disadvantage because of the high resistance of the bony ankle and wrist (>50% of whole body resistance) included in the measurement, although hands and feet contribute very little (1.8%) to body weight.^24^ Our results show that the goodness of fit for BIA equations using the conventional 8-adhesive electrodes in the supine position is not superior to the standing position. This may be due to the fact that body compartments and anatomic regions are all highly correlated with each other. However, our models have been trained with the data and therefore goodness of fit only gives information how well this model can ‘predict’ data points that are already used to estimate its parameters. Because predictive accuracy reveals how well a model can predict new data points, we applied our models to the cross-validation sample and found that predictive power of plate vs gel electrode protocols was similar for all outcome parameters (pure error for plate electrode protocols ranged between 0.26 for the arms and 2.3 for the whole body; for adhesive gel electrodes, it ranged between 0.28 for the arms and 2.1 for the whole body, respectively).

On the other hand, we found a significantly better prediction using segmental impedance measurements instead of a conventional wrist-ankle technology. The regional distribution of extracellular water changes from supine to standing position towards an increase in extracellular water in the limbs and a corresponding decrease in the trunk.^25^ In contrast to conventional impedance from wrist to ankle at one body side only, segmental impedance measurement is thought to be less sensitive to changes in body position, because it is not based on the assumption that the body (limbs and trunk) resembles a uniform cylinder with a constant relationship between mean cross-sectional areas and a homogeneous water distribution. There are however reports that segmental impedance measurements are less accurate in predicting individual limb LST_DXA_ than whole-body impedance^26^ and early studies that estimated limb SMM_MRI_ by segmental BIA measurements found a low bias but large limits of agreement that indicate a low precision of the method.^27, 28^ In these studies segmental impedance was however measured using adhesive electrodes attached to the ipsilateral wrist, the shoulder; the upper iliac spine and the ankle. As a main drawback, this approach is cumbersome and error prone because it requires the correct placement of electrodes at anatomical landmarks of the trunk. Modern tetrapolar impedance devices like the seca mBCA 525 and 515 enable segmental impedance measurement of the right and left arm, the trunk as well as the right and left leg by using four electrodes on each side of the body at the hands and feet. The selection or switching between the individual detecting and source electrodes allow the limbs to be used as virtual electrodes that measure the opposite side of the body.^29^ Differences compared with electrode placement at the shoulder and the upper iliac spine are <2%.^30^

Limits of agreement of the bias between BIA and reference method were also calculated as a percentage of the mean reference value (LOA% Table 4). These values reveal that the predictive accuracy of BIA compared with MRI as a reference is clinically acceptable when whole-body SMM was assessed (between 11 and 12% for different ethnicities) but it becomes limited when small compartments of the body are assessed (e.g. it ranged between 20 and 29% for the arms). Discrepancies in the assumptions of the homogeneous bioelectrical model that lead to a higher measurement error not only occur with changes in body position but also with differences in body shape that are associated with aging (decreasing limb relative to trunk diameter), obesity (apple and pear shape of body fat distribution) and ethnic differences (in trunk relative to leg length and regional adiposity and muscularity). Segmental BIA was therefore used to develop two indices, Index*R*_50 trunk/extremities_ and IndexXc_50 trunk/extremities_ that represent the relative contribution of trunk and extremities to total body conductivity and help to correct for differences in body shape (these indices explained 0.4% of the variance in total SMM_MRI_ (Table 5)).

Validation of the BIA equations predicting total and limb SMM and LST in a multiethnic population with a great variance in muscularity has shown that BIA results correctly identified ethnic differences in muscularity and visceral adiposity (Table 4). However, the comparison of bias between Caucasians and different ethnic groups revealed some ethnical effects on the accuracy of BIA prediction equations that confirm previous results of other authors who used a more simple non-segmental and non-phase-sensitive (without Xc output) impedance measurement.^17^ Because Xc is used to calculate phase angle (phase angle=arctan(Xc/*R*) × (180/*π*)) and is related to body cell mass and soft tissue composition, it is therefore expected to improve the prediction of SMM. Reactance contributed 1.5–8.3% to the variability in SMM and appendicular LST prediction (Table 5). Although statistically significant, the differences of BIA biases between Caucasians and other ethnicities are small (Table 4) and support our previous findings that a BIA equation is more device specific than population specific.^18^ The current software of the seca mBCA 515 and 525 applies ethnic-specific correction factors to equations for prediction of SMM and VAT (personal communication by the manufacturer) that were not used for the calculation of results in the present analyses (Table 4). As a limitation to our study, the significant bias of all BIA results for SMM and LST in Caucasians investigated in New York suggests an additional impact of discrepancies in reference methods between the labs in Kiel and New York (i.e. Hologic vs GE DXA scanner and Siemens vs GE MRI machines).

Although both seca BIA devices are able to measure a spectrum of frequencies, the single 50 kHz frequency is used for SMM and VAT prediction because no improvement of results was obtained using bioelectrical impedance spectroscopy (unpublished results). This is in line with a recent report showing that despite a higher accuracy of bioelectrical impedance spectroscopy methods over 50 kHz, the magnitude of the improvement was rather small for both prediction at population and individual level.^31^

Finally, the differences between LST_DXA_ and SMM_MRI_ at the arms and legs were considerable, higher in men compared with women and more pronounced in obese participants (see results) due to a higher contribution of connective tissue to total lean mass. This leads to a sizable overestimation of SMM with increasing age and adiposity when predicting appendicular LST_DXA_ instead of SMM_MRI_. Comparison of DXA with the results of imaging technology (computer tomography) has shown a 5–8% lower SMM at the limbs when compared with appendicular LST_DXA_.^32^ Since the differentiation between SMM and the lean compartment of connective tissue may be difficult using the electric properties of the tissue, a validation of the prediction of SMM by BIA is required in severely obese and/or elderly people.

In conclusion, a high accuracy of phase-sensitive segmental BIA was observed with no difference in goodness of fit between standing and supine positions. Segmental measurements improved the prediction of whole-body compartments compared with conventional wrist-to-ankle measurements. A correction factor for certain ethnicities may be required. BIA equations based on MRI as a reference are more accurate for prediction of SMM when compared with DXA.

## Figures and Tables

**Table 1:** Basic characteristics of the Caucasian study population for generation of BIA equations and of the multiethnic study population used to cross-validate these equations

	*Female,* n*=66*	*Male,* n*=70*	*All,* n*=136*
*Equation generation population*			
Age (years)	40.5±12.8	39.6±12.0	40.0±12.3
BMI (kg/m^2^)	23.9±3.5	26.0±3.3^***^	25.0±3.5
Waist circumference (cm)	82.0±10.8	92.4±10.1^***^	87.3±11.6
Ht^2^/*R*_50_ (cm^2^/Ω)	48.2±6.3	70.3±8.5^***^	59.6±13.4
*R*_50_ (Ω)	588±50	468±50^***^	526±78
Xc_50_ (Ω)	60.0±6.6	56.7±7.0^**^	58.3±7.0
Index *R*_50_	0.0907±0.0082	0.1040±0.0095^***^	0.0975±0.0111
Index Xc_50_	0.1056±0.0095	0.1240±0.0101^***^	0.1151±0.0135
			
*Cross-validation population*			
Age (years)	39.9±12.5	40.4±13.2	40.2±12.8
BMI (kg/m^2^)	25.0±3.8	25.8±4.2	25.4±4.0
Waist circumference (cm)	83.1±11.2	90.1±12.7^**^	86.6±12.4
Ht^2^/*R*_50_ (cm^2^/Ω)	44.6±5.3	62.7±9.2^***^	53.6±11.8
*R*_50_ (Ω)	595±60	492±60^***^	544±79
Xc_50_ (Ω)	62.7±7.2	58.5±8.5^**^	60.6±8.1
Index *R*_50_	0.0955±0.0129	0.1051±0.0124^***^	0.1003±0.0135
Index Xc_50_	0.0971±0.0125	0.1194±0.0111^***^	0.1082±0.0163

Abbreviations: BMI, body mass index; Ht, height.

*P<0.05, **P<0.01, ***P<0.001 sex difference by *t*-test. Impedance raw data were obtained in the supine position with eight electrodes and averaged for the left and right side of the body.

**Table 2:** Body composition obtained by MRI or DXA as a reference and predicted by BIA obtained in a standing and lying position with ‘8-electrode’ segmental or conventional wrist-to-ankle technique in the Caucasian population. Goodness of fit of BIA equations was assessed by *R* ^2^ and precision by the PE

	*Mean*±*s.d.*	*PE*	R^*2*^
*MRI*			
SMM total body (kg)	26.8±6.9		
SMM arms (kg)	3.41±1.09		
SMM legs (kg)	11.46±2.59		
VAT (l)	1.6±1.4		
			
*DXA*			
LST arms (kg)	6.00±1.95		
LST legs (kg)	18.07±3.92		
			
*BIA standing segmental*			
SMM total body (kg)	26.8±6.8	1.2	0.97
SMM arms (kg)	3.41±1.07	0.27	0.94
SMM legs (kg)	11.45±2.45	0.76	0.91
VAT (l)	1.6±1.2	0.6	0.81
LST arms (kg)	6.04±1.93	0.42	0.95
LST legs (kg)	18.15±3.88	0.81	0.96
			
*BIA supine segmental*			
SMM total body (kg)	26.8±6.8	1.2	0.97
SMM arms (kg)	3.41±1.06	0.26	0.94
SMM legs (kg)	11.46±2.47	0.73	0.92
VAT (l)	1.6±1.2	0.6	0.80
LST arms (kg)	5.99±1.90	0.42	0.95
LST legs (kg)	18.07±3.81	0.83	0.95
			
*BIA supine wrist–ankle*			
SMM total body (kg)	26.7±6.7	1.3	0.96**
VAT (l)	1.6±1.2	0.6	0.79

Abbreviations: BIA, bioelectrical impedance analysis; BMI, body mass index; DXA, dual X-ray absorptiometry; LST, lean soft tissue; MRI, magnetic resonance imaging; PE, pure error; R^2^, determination coefficient; SMM, skeletal muscle mass; VAT, visceral adipose tissue.

^**^P<0.01, significant difference in correlation from supine segmental BIA.

**Table 3:** Basic characteristics of the multiethnic validation population (*n*=123)

	*Female*	*Male*	*All*
*Caucasians*	n*=16*	n*=16*	n*=32*
Age (years)	42.7±13.7	43.1±15.7	42.9±14.5
BMI (kg/m^2^)	25.2±4.2	26.8±4.6	26.0±4.4
Waist circumference (cm)	85.2±9.9	94.8±14.3*	90.0±13.0
			
*Asians*	n*=17*	n*=14*	n*=31*
Age (years)	39.4±12.2	38.3±13.2	38.9±12.5
BMI (kg/m^2^)	22.5±1.9	23.4±3.6	22.9±2.8
Waist circumference (cm)	76.5±8.9	82.5±11.0	79.2±10.2
			
*Afro-Americans*	n*=14*	n*=16*	n*=30*
Age (years)	36.1±10.2	40.9±11.7	38.7±11.1
BMI (kg/m^2^)	24.6±3.8	26.0±3.8	25.3±3.8
Waist circumference (cm)	81.8±13.4	87.7±10.7	84.9±12.2
			
*Hispanics*	*n*=15	*n*=15	*n*=30
Age (years)	41.1±13.6	39.0±12.5	40.0±12.9
BMI (kg/m^2^)	28.2±2.8	26.7±4.2	27.5±3.6
Waist circumference (cm)	89.7±9.0	94.7±10.9	92.2±10.2

Abbreviation: BMI, body mass index.

*P<0.05, sex differences within ethnic group by *t*-test.

**Table 4:** Validity of SMM, visceral fat and LSTpredicted from BIA equations vs respective results from MRI and DXA compared between different ethnic groups

	*SMM, VAT or LST measured by MRI or DXA*	*SMM, VAT or LST predicted by BIA*	*Bias*^a^ *(mean*±*2* *s.d.)*	*LOA*^b^ *%*	*Correlation coefficient*^c^ *r*
*Caucasians (*n*=32)*					
SMM total body (kg)	23.3±6.2	24.5±5.9^†††^	1.2±2.6	11	0.05
SMM arms (kg)	3.11±1.06	2.95±0.85^†^	−0.17±0.69	22	0.39^‡‡‡^
SMM legs (kg)	9.90±2.40	10.97±2.17^†††^	1.07±2.03	20	0.05
LST arms (kg)	5.87±1.84	5.17±1.62^†††^	−0.70±1.10	19	0.16^‡^
LST legs (kg)	16.70±3.59	17.44±3.43^††^	0.74±2.32	14	0.02
VAT (l)	2.2±1.7	2.0±1.7	−0.1±1.1	49	0.01
					
*Asians (*n*=31)*					
SMM total body (kg)	19.7±5.1*	21.3±5.7^†††^	1.6±2.2	11	0.35^‡‡‡^
SMM arms (kg)	2.59±0.93*	2.56±0.83	−0.03±0.57	22	0.12
SMM legs (kg)	8.48±1.89*	9.79±2.05^†††^	1.31±1.36	16	0.06
LST arms (kg)	5.01±1.60	4.44±1.56^†††^	−0.57±0.52	10	0.03
LST legs (kg)	14.59±2.68*	15.56±3.07^†††^	0.97±1.57	11	0.25^‡‡^
VAT (l)	1.9±1.1	1.2±0.7^†††^	−0.7±1.1***	59	0.44^‡‡‡^
					
*Afro-Americans (*n*=30)*					
SMM total body (kg)	26.1±7.1	26.4±6.4	0.3±3.2*	12	0.18^‡^
SMM arms (kg)	3.64±1.21	3.25±0.92^†††^	−0.38±1.04	29	0.32^‡‡^
SMM legs (kg)	11.39±2.72*	11.45±2.47	0.06±1.64***	14	0.09
LST arms (kg)	7.17±2.12*	5.67±1.74^†††^	−1.51±1.33***	19	0.32^‡‡^
LST legs (kg)	18.70±4.36	18.16±3.82^†^	−0.54±2.26***	12	0.23^‡‡^
VAT (l)	1.2±1.1**	1.3±1.2	0.1±1.3	104	0.04
					
*Hispanics (*n*=30)*					
SMM total body (kg)	21.5±5.3	23.2±5.4^†††^	1.7±2.3	11	0.00
SMM arms (kg)	2.85±0.86	2.75±0.79	−0.10±0.58	20	0.08
SMM legs (kg)	8.51±1.77*	10.45±2.04^†††^	1.94±2.20**	26	0.06
LST arms (kg)	5.68±1.44	4.87±1.50^†††^	−0.80±0.82	14	0.02
LST legs (kg	15.77±2.87	16.51±3.16^†††^	0.74±2.16	14	0.07
VAT (l)	3.1±1.8*	2.2±1.2^†††^	−0.9±2.5**	81	0.21^‡^

Abbreviations: BIA, bioelectrical impedance analysis; DXA, dual X-ray absorptiometry; LOA, limits of agreement; LST, lean soft tissue; MRI, magnetic resonance imaging; SMM, skeletal muscle mass; VAT, visceral adipose tissue.

*P<0.05, **P<0.01 and ***P<0.001 significantly different from Caucasians by *t*-test.

^†^*P*<0.05, ^††^*P*<0.01 and ^†††^*P*<0.001 significantly different from reference (MRI or DXA) by paired *t*-test.

^‡^*P*<0.05, ^‡‡^*P*<0.01 and ^‡‡‡^*P*<0.001 significant correlation.

Results of the LOA analysis are given as bias and 95% limits of agreement (±2 s.d.) for SMM, and LST and VAT measured by reference method and compared with prediction by BIA.

a Bias was calculated as result obtained from BIA measurement minus reference method (MRI, DXA). 95% Limits of agreement was calculated as±2 s.d.

b LOA are given as a percentage of mean value measured by the reference method.

c Correlation was calculated as Pearson’s correlation coefficient for the relationship between (result_reference method_+result_BIA_)/2 and the bias.

**Table 5:** Results of six stepwise regression analyses for measurements with eight electrodes in supine position using data from the Caucasian population in Kiel with total and regional SMM_MRI_ (kg) and VAT_MRI_ (l) as the dependent variables

*Predictor*	R^*2*^	*SEE*	P*-value*	*VIF*
*SMM total body (kg)*				
Ht^2^/*R*_50 right and left side_ (Ω)	0.912	2.037	*P*<0.0001	15.5
Xc_50 right and left side_ (Ω)	0.950	1.543	*P*<0.0001	2.9
Weight (kg)	0.962	1.359	*P*<0.0001	3.5
Gender	0.965	1.303	*P*<0.01	5.8
Index *R*_50 trunk/extremities_	0.969	1.235	*P*<0.0001	3.5
Index Xc_50 trunk/extremities_	0.969	1.225	*P*=0.01	5.0
*R*_50 right and left side_ (Ω)	0.971	1.201	*P*=0.01	10.6
Intercept			*P*=0.13	
				
*SMM right arm (kg)*				
Ht^2^/*R*_50 right arm_ (Ω)	0.901	0.175	*P*<0.0001	9.4
Xc_50 right arm_ (Ω)	0.916	0.162	*P*<0.01	2.3
Gender	0.917	0.162	*P*=0.03	4.4
Index *R*_50 trunk/extremities_	0.919	0.160	*P*=0.08	1.9
Weight (kg)	0.921	0.159	*P*=0.10	2.6
Age (years)	0.921	0.160	*P*=0.35	1.2
Intercept			*P*=0.32	
				
*SMM left arm (kg)*				
Ht^2^/*R*_50 left arm_ (Ω)	0.901	0.171	*P*<0.0001	7.9
Xc_50 left arm_ (Ω)	0.919	0.156	*P*<0.01	2.1
Gender	0.926	0.149	*P*<0.0001	4.1
Index *R*_50 trunk/extremities_	0.933	0.142	*P*<0.001	1.8
Weight (kg)	0.936	0.139	*P*<0.01	2.5
Age (years)	0.939	0.137	*P*=0.03	1.2
Intercept			*P*=0.29	
				
*SMM right leg (kg)*				
Ht^2^/*R*_50 right leg_ (Ω)	0.800	0.582	*P*<0.0001	8.4
Xc_50 right leg_ (Ω)	0.856	0.497	*P*<0.001	2.8
Weight (kg)	0.882	0.451	*P*<0.0001	3.5
Gender	0.891	0.434	*P*<0.001	3.8
Index *R*_50 trunk/extremities_	0.899	0.419	*P*<0.01	3.1
Age (years)	0.905	0.410	*P*=0.01	1.4
Index Xc_50 trunk/extremities_	0.906	0.408	*P*=0.11	4.4
Intercept			*P*=0.84	
				
*SMM left leg (kg)*				
Ht^2^/*R*_50 left leg_ (Ω)	0.793	0.589	*P*<0.0001	9.2
Xc_50 left leg_ (Ω)	0.876	0.457	*P*<0.0001	3.1
Weight (kg)	0.899	0.414	*P*<0.0001	3.6
Index R_50 trunk/extremities_	0.904	0.405	*P*<0.001	3.2
Index Xc_50 trunk/extremities_	0.913	0.388	*P*<0.01	4.7
Age (years)	0.916	0.383	*P*=0.03	1.3
Gender	0.917	0.383	*P*=0.30	4.4
Intercept			*P*=0.01	
				
*VAT (l)*				
Waist (cm)	0.666	0.793	*P*<0.001	NC
Waist^2^ (cm^2^)	0.691	0.765	*P*<0.0001	NC
Waist^2^ × gender (cm^2^)	0.727	0.722	*P*<0.001	NC
Ht^2^^2^/*R*_50 trunk_ (Ω)	0.774	0.659	*P*<0.0001	NC
Age (years)	0.790	0.638	*P*<0.01	NC
Waist × gender (cm)	0.798	0.628	*P*<0.01	NC
Height (cm)	0.805	0.620	*P*=0.03	NC
Intercept			*P*=0.38	

Abbreviations: LST, lean soft tissue; MRI, magnetic resonance imaging; NC, not calculated because the regression for VAT prediction is nonlinear; R^2^, determination coefficient; SEE, standard error of the estimate; SMM, skeletal muscle mass; VAT, visceral adipose tissue; VIF, variance inflation factors.

## References

[bib1] Bosy-Westphal A, Müller MJ. Identification of skeletal muscle mass depletion across age and BMI groups in health and disease—there is need for a unified definition. Int J Obes (Lond) 2015; 39: 379–386. 25174451 10.1038/ijo.2014.161

[bib2] Kim JH, Choi SH, Lim S, Kim KW, Lim JY, Cho NH et al. Assessment of appendicular skeletal muscle mass by bioimpedance in older community-dwelling Korean adults. Arch Gerontol Geriatr 2014; 58: 303–307. 24309033 10.1016/j.archger.2013.11.002

[bib3] Kim M, Kim H. Accuracy of segmental multi-frequency bioelectrical impedance analysis for assessing whole-body and appendicular fat mass and lean soft tissue mass in frail women aged 75 years and older. Eur J Clin Nutr 2013; 67: 395–400. 23388666 10.1038/ejcn.2013.9

[bib4] Kim M, Shinkai S, Murayama H, Mori S. Comparison of segmental multifrequency bioelectrical impedance analysis with dual-energy X-ray absorptiometry for the assessment of body composition in a community-dwelling older population. Geriatr Gerontol Int 2015; 15: 1013–1022. 25345548 10.1111/ggi.12384

[bib5] Kyle UG, Genton L, Hans D, Pichard C. Validation of a bioelectrical impedance analysis equation to predict appendicular skeletal muscle mass (ASMM). Clin Nutr 2003; 22: 537–543. 14613755 10.1016/s0261-5614(03)00048-7

[bib6] Pietiläinen KH, Kaye S, Karmi A, Suojanen L, Rissanen A, Virtanen KA. Agreement of bioelectrical impedance with dual-energy X-ray absorptiometry and MRI to estimate changes in body fat, skeletal muscle and visceral fat during a 12-month weight loss intervention. Br J Nutr 2013; 109: 1910–1916. 22935366 10.1017/S0007114512003698

[bib7] Rangel-Peniche DB, Raya-Giorguli G, Alemán-Mateo H. Accuracy of a predictive bioelectrical impedance analysis equation for estimating appendicular skeletal muscle mass in a non-Caucasian sample of older people. Arch Gerontol Geriatr 2015; 61: 39–43. 25857600 10.1016/j.archger.2015.03.007

[bib8] Tanaka NI, Hanawa S, Murakami H, Cao ZB, Tanimoto M, Sanada K et al. Accuracy of segmental bioelectrical impedance analysis for predicting body composition in pre- and postmenopausal women. J Clin Densitom 2015; 18: 252–259. 25174687 10.1016/j.jocd.2014.07.002

[bib9] Villani AM, Miller M, Cameron ID, Kurrle S, Whitehead C, Crotty M. Body composition in older community-dwelling adults with hip fracture: portable field methods validated by dual-energy X-ray absorptiometry. Br J Nutr 2013; 109: 1219–1229. 22914101 10.1017/S0007114512003170

[bib10] Xu L, Cheng X, Wang J, Cao Q, Sato T, Wang M et al. Comparisons of body-composition prediction accuracy: a study of 2 bioelectric impedance consumer devices in healthy Chinese persons using DXA and MRI as criteria methods. J Clin Densitom 2011; 14: 458–464. 21835660 10.1016/j.jocd.2011.04.001

[bib11] Yoshida D, Shimada H, Park H, Anan Y, Ito T, Harada A et al. Development of an equation for estimating appendicular skeletal muscle mass in Japanese older adults using bioelectrical impedance analysis. Geriatr Gerontol Int 2014; 14: 851–857. 24450604 10.1111/ggi.12177

[bib12] Schutz Y, Kyle UUG, Pichard C. Fat-free mass index and fat mass index percentiles in Caucasians aged 18–98 y. Int J Obes 2002; 26: 953–960. 10.1038/sj.ijo.0802037 12080449

[bib13] Addison O, Marcus RL, Lastayo PC, Ryan AS. Intermuscular fat: a review of the consequences and causes. Int J Endocrinol 2014; 309570. 24527032 10.1155/2014/309570 PMC3910392

[bib14] Schautz B, Later W, Heller M, Muller MJ, Bosy-Westphal A. Total and regional relationship between lean and fat mass with increasing adiposity—impact for the diagnosis of sarcopenic obesity. Eur J Clin Nutr 2012; 66: 1356–1361. 23031852 10.1038/ejcn.2012.138

[bib15] Wang Z, Deurenberg P, Heymsfield SB. Cellular-level body composition model. A new approach to studying fat-free mass hydration. Ann NY Acad Sci 2000; 904: 306–311. 10865761 10.1111/j.1749-6632.2000.tb06472.x

[bib16] Bosy-Westphal A, Later W, Hitze B, Sato T, Kossel E, Gluer CC et al. Accuracy of bioelectrical impedance consumer devices for measurement of body composition in comparison to whole body magnetic resonance imaging and dual X-ray absorptiometry. Obes Facts 2008; 1: 319–324. 20054195 10.1159/000176061 PMC6452160

[bib17] Janssen I, Heymsfield SB, Baumgartner RN, Ross R. Estimation of skeletal muscle mass by bioelectrical impedance analysis. J Appl Physiol 2000; 89: 465–471. 10926627 10.1152/jappl.2000.89.2.465

[bib18] Bosy-Westphal A, Schautz B, Later W, Kehayias JJ, Gallagher D, Muller MJ. What makes a BIA equation unique? Validity of eight-electrode multifrequency BIA to estimate body composition in a healthy adult population. Eur J Clin Nutr 2013; 67 (Suppl 1), S14–S21. 23299866 10.1038/ejcn.2012.160

[bib19] Gallagher D, Kelley DE, Yim JE, Spence N, Albu J, Boxt L et al. Adipose tissue distribution is different in type 2 diabetes. Am J Clin Nutr 2009; 89: 807–814. 19158213 10.3945/ajcn.2008.26955 PMC2714397

[bib20] Song MY, Ruts E, Kim J, Janumala I, Heymsfield S, Gallagher D. Sarcopenia and increased adipose tissue infiltration of muscle in elderly African American women. Am J Clin Nutr 2004; 79: 874–880. 15113728 10.1093/ajcn/79.5.874

[bib21] Snyder WS, Cook MJ, Nasset ES, Karhausen LR, Howells GP, Tipton IH. Report of the Task Group on Reference Man. Pergamon Press: Oxford, UK, 1975.

[bib22] Eid M, Gollwitzer M, Schmitt M. Statistik und Forschungsmethoden. 4th edn, Beltz: Basel, Switzerland, 2013, p 578.

[bib23] Bland JM. Statistical methods for assessing agreement between two methods of clinical measurement. Lancet 1986; 1: 307–310. 2868172

[bib24] Croney J. Anthropometry for Designers. Van Nostrand Reinhold: New York, NY, USA, 1981 pp 12–13.

[bib25] Zhu F, Schneditz D, Wang E, Levin NW. Dynamics of segmental extracellular volumes during changes in body position by bioimpedance analysis. J Appl Physiol 1998; 85: 497–504. 9688726 10.1152/jappl.1998.85.2.497

[bib26] Sergi G, De Rui M, Veronese N, Bolzetta F, Berton L, Carraro S et al. Assessing appendicular skeletal muscle mass with bioelectrical impedance analysis in free-living Caucasian older adults. Clin Nutr 2015; 34: 667–673. 25103151 10.1016/j.clnu.2014.07.010

[bib27] Elia M, Fuller NJ, Hardingham CR, Graves M, Screaton N, Dixon AK et al. Modeling leg sections by bioelectrical impedance analysis, dual-energy X-ray absorptiometry, and anthropometry: assessing segmental muscle volume using magnetic resonance imaging as a reference. Ann NY Acad Sci 2000; 904: 298–305. 10865760 10.1111/j.1749-6632.2000.tb06471.x

[bib28] Fuller NJ, Graves M, Screaton N, Dixon AK, Ward LC, Elia M. Predicting composition of leg sections with anthropometry and bioelectrical impedance analysis, using magnetic resonance imaging as reference. Clin Sci (Lond) 1999; 96: 647–657. 10334971

[bib29] Organ LW, Bradham GB, Gore DT, Lozier SL. Segmental bioelectrical impedance analysis: theory and application of a new technique. J Appl Physiol 1994; 77: 98–112. 7961281 10.1152/jappl.1994.77.1.98

[bib30] Cornish BH, Jacobs A, Thomas BJ, Ward LC. Optimizing electrode sites for segmental bioimpedance measurements. Physiol Meas 1999; 20: 241–259. 10475578 10.1088/0967-3334/20/3/302

[bib31] Seoane F, Abtahi S, Abtahi F, Ellegård L, Johannsson G, Bosaeus I et al. Mean expected error in prediction of total body water: a true accuracy comparison between bioimpedance spectroscopy and single frequency regression equations. Biomed Res Int 2015; 656323. 26137489 10.1155/2015/656323 PMC4468285

[bib32] Wang W, Wang Z, Faith MS, Kotler D, Shih R, Heymsfield SB. Regional skeletal muscle measurement: evaluation of new dual-energy X-ray absorptiometry model. J Appl Physiol 1999; 87: 1163–1171. 10484591 10.1152/jappl.1999.87.3.1163

